# Integrin-Mediated Adhesion Promotes Centrosome Separation in Early Mitosis

**DOI:** 10.3390/cells11081360

**Published:** 2022-04-16

**Authors:** Siamak A. Kamranvar, Deepesh Kumar Gupta, Anishia Wasberg, Liangwen Liu, Joan Roig, Staffan Johansson

**Affiliations:** 1 Department of Medical Biochemistry and Microbiology (IMBIM), Biomedical Center, Uppsala University, 75123 Uppsala, Sweden; siamak.kamranvar@imbim.uu.se (S.A.K.); anishia.wasberg@imbim.uu.se (A.W.); 2 Department of Pediatrics, Washington University School of Medicine, St. Louis, MO 63110, USA; gupta.d@wustl.edu; 3 Department of Medical Cell Biology (MCB), Biomedical Center, Uppsala University, 75123 Uppsala, Sweden; liangwen.liu@mcb.uu.se; 4 Cell Signaling Group, Molecular Biology Institute of Barcelona (IBMB-CSIC), 08028 Barcelona, Spain; joan.roig@ibmb.csic.es

**Keywords:** integrin, FAK, PLK1, centrosome, mitosis, Eg5

## Abstract

Integrin-mediated adhesion to the extracellular matrix is a key regulator of the cell cycle, as demonstrated for the passage of the G1/S checkpoint and the completion of cytokinetic abscission. Here, integrin-dependent regulation of the cell cycle in G2 and early M phases was investigated. The progression through the G2 and M phases was monitored by live-cell imaging and immunofluorescence staining in adherent and non-adherent fibroblast cells. Non-adherent cells, as well as adherent cells lacking FAK activity due to suppressed expression or pharmacological inhibition, exhibited a prolonged G2 phase and severely defect centrosome separation, resulting in delayed progress through the early mitotic stages. The activation of the critical mitotic regulator PLK1 and its indirect target Eg5, a kinesin-family motor protein driving the centrosome separation, were reduced in the cells lacking FAK activity. Furthermore, the absence of integrin adhesion or FAK activity destabilized the structural integrity of centrosomes and often caused detachment of pericentriolar material from the centrioles. These data identify a novel adhesion-dependent mechanism by which integrins via FAK and PLK1 contribute to the regulation of the cell cycle in the G2 and early M phases, and to the maintenance of genome integrity.

## 1. Introduction

Cell proliferation and cell adhesion to the extracellular matrix (ECM) are closely interdependent processes. Integrin adhesion receptors sense the composition and mechanical properties of the ECM and transmit the information via several signaling pathways to modify the cell behavior, including proliferation, differentiation, and migration [1]. The ligand-induced receptor clustering leads to the recruitment of intracellular proteins to form focal adhesion complexes in which FAK is auto-phosphorylated at Tyr397 [2]. Src-family kinases are activated after docking to pTyr397-FAK, and then phosphorylate FAK on other tyrosine residues required for full FAK catalytic activity and adaptor functions. The FAK-Src complex phosphorylates several downstream targets to initiate a cascade of signaling pathways [3]. The focal adhesion complexes undergo dramatic modulation during the cell cycle. They grow in size and number during the S phase driven by cyclin A-CDK1 activity, but shrink in the G2 phase and dissolve to a large extent when the cells transiently round-up at the mitotic entry [4,5,6]. The rounded cells remain attached to the ECM via reticular adhesions, which contain integrin αVβ5 but lack FAK and other focal adhesion proteins at the contact sites [7]; in addition, varying amounts of focal adhesion remnants with altered composition were reported to be present during the rounded stage in different cell types [7,8,9,10]. 

The mechanisms by which the adhesion complexes contribute to cell proliferation are best understood for the passage of the G1/S checkpoint and the completion of cytokinesis [11,12]. Integrins via downstream FAK signaling pathways upregulate cyclin D and downregulate the CDKs inhibitors p21 and p27, and thereby contribute to G1/S transition [13,14,15]. Cytokinesis starts during anaphase and proceeds sequentially through several stages, including cleavage furrow ingression, the midbody (MB) formation, and eventually the abscission [16,17]. Integrin-induced FAK signaling is involved only in the final step of the cytokinesis process, abscission, by regulating PLK1-mediated recruitment of centrosomal protein 55 (Cep55) to the MB [18]. The timely Cep55 localization to the MB is needed for the subsequent recruitment of endosomal sorting complexes required for transport (ESCRTs) to fulfill the abscission [18,19].

PLK1 is a mitotic master kinase that plays a major role in the execution of diverse mitotic events in a coordinated manner [20]. PLK1 is required for the activation of the cyclin B-CDK1 complex, whose kinase activity is needed for mitotic entry and the cell cycle progression through the early mitotic stages [21,22]. PLK1 is also involved in the maturation of centrosomes, the main microtubule organizing centers (MTOCs) required for the formation of the mitotic spindle in most animal cells [23,24]. After normal cell division, each cell contains a single centrosome composed of a pair of centrioles embedded in the pericentriolar material (PCM), and after duplication during S phase, the protein filaments linking the two centrosomes are dissociated due to phosphorylations by PLK1 and NEK2 in the late G2 phase [25,26]. The translocation of the centrosomes during early mitosis to opposite sides of the nucleus depends on the action of microtubule-based motor proteins such as dynein and kinesins, and the actomyosin network [27]. PLK1 induces phosphorylation of the mitotic kinesin Eg5 (KIF11) at Ser1033 via NEKs 6 and 7 and at Thr926 via CDK1, whereby Eg5 is activated and generates the main force leading to the centrosome translocation to form a bipolar mitotic spindle [28]. Centrosome defects may increase the incidence of multipolar mitosis and incorrectly attached chromosomes to microtubules, leading to chromosomal miss-segregation and aneuploidy, which is the predominant type of genomic instability found in human cancers [29]. Chromosomal mis-segregation can also cause mitotic cell death or blocked cytokinesis due to the formation of lagging chromosomes [30].

Cell adhesion to ECM is known to affect several aspects of centrosome functions. Integrin-mediated cell adhesion has been observed to influence the planar orientation of the mitotic spindle [31,32]. Moreover, the mutation in the β1 integrin cytoplasmic domain was shown to cause the formation of multipolar mitotic spindles and reduced the nucleation of microtubules at the interphase centrosome [33,34]. In β1 integrin null astrocytes, the interphase centrosome was fragmented and failed to promote cell polarization for the directed cell migration [35]. 

Here, we found for the first time that integrin-mediated adhesion supports centrosome separation during the G2/early M phase. This function was found to be FAK signaling-dependent and associated with PLK1 activity, whose absence resulting in low Eg5 activity, monopolar spindle formation, and cells stuck in the early mitosis. Moreover, the mitotic centrosomes were less stable in the absence of integrin adhesion or FAK activity as revealed by the presence of PCM fragments detached from the centrioles in a significant number of the cells.

## 2. Materials and Methods

### 2.1. Cell Lines and Culturing of Mitotic Cells

Human non-transformed fibroblast cells (hTERT-immortalized BJ cells, accession number CVCL_3653, obtained from Ludwig Institute, Uppsala, Sweden) and Tet-FAK MEF (FAK-null MEF cell line, accession number CRL-2644, with tetracycline-regulated expression of FAK received from SK Hanks [36]) were cultured in the complete medium of Dulbecco’s modified Eagle (DMEM, Gibco, Life Technologies, Bleiswijk, The Netherlands) supplemented with 10% fetal bovine serum (FBS, FB-1090-500, Werner Saveen, Limhamn, Sweden), 100 U/mL penicillin, and 0.1 mg/mL streptomycin. The cells were kept at 37 °C in a humidified atmosphere containing 5% CO_2_. Tet-FAK cells were cultured in the complete medium containing 1 μg/mL doxycycline to suppress the FAK expression when needed. FAK expression was essentially abolished after 3 days of treatment with doxycycline. 

### 2.2. Cell Synchronization

The cells were synchronized in the early S phase using a double thymidine block in which they were treated with 2 mM thymidine for 18 h followed by 9 h release and then the same treatment again for 17 h. After the second thymidine release, the synchronized cells were cultured for 5 h in the complete medium before trypsinizing and splitting into adhesive or non-adhesive plates.

To synchronize the cells in early mitosis, they were treated with nocodazole (20 ng/mL) for 5 h as described in the figure legends. The synchronized cells were then collected by the shake-off method [37], in which the loosely attached mitotic cells were detached by tapping the culture flasks. To release mitotic cells from the nocodazole block, the cells were washed twice with pre-warmed PBS and once with the complete medium (approximately 20 min in total). The cells were then cultured in either bacterial plates coated with Pluronic (10 mg/mL, F108 NF Prill Poloxamer 338, D-BASF, Monheim, Germany) for the adhesion-independent condition (suspension), or in cell culture plates coated with fibronectin (40 μg/mL) for the adhesion-dependent condition. PLL (Poly-L-Lysine, P9155, Sigma, Saint Louis, MO, USA)-coated plates were also used for the conditioning of integrin-independent adhesion to chase the cells with live imaging. Where indicated, the cells were treated with the FAK inhibitor PF-562271 (5 μM, 2B Scientific, Bicester, UK), the PLK1 inhibitor BI-6727 (2 μM, MedChemExpress, Monmouth Junction, NJ, USA), or the Aurora A inhibitor MNL-8237 (0.5 μM, MedChemExpress, Monmouth Junction, NJ, USA). Two other FAK inhibitors, PND1186 (Cayman Chemicals, Ann Arbor, MI, USA) and FAK inhibitor 14 (TOCRIS, Bristol, UK), were also used to confirm the results obtained with the PF-562271.

### 2.3. Live-Cell Imaging

Live-cell imaging was performed using an inverted microscope (Nikon-Eclipse Ti-U, Melville, NY, USA) equipped with a CCD camera (Andor’s multi pixel sCMOS camera, Oxford Instruments, Abingdon, UK) and a cell culture chamber with a constant supply of humidified 5% CO_2_ and temperature control. The collected mitotic cells were replated in the fibronectin- or PLL-coated culture plates and monitored for the indicated time periods. The images were acquired in 5 to 15 min time intervals using a 20× magnification objective and phase contrast filter of the time-lapse microscope. DNA was labeled using either SiR-DNA or SPY-DNA (Spirochrome) with a concentration of 0.5 mM. 

### 2.4. Immunofluorescence Staining and Quantification of pPLk1 Signal Intensity

For the adherent condition, the mitotic cells were cultured on fibronectin-coated coverslips, whereas they were cultured in the Pluronic-coated plates for the non-adherent condition and thereafter deposited on glass slides by cytospin centrifugation. Subsequently, the cells were fixed by cold methanol at −20 °C for 20 min and then washed twice in PBS for 5 min. After incubation in blocking buffer, PBS containing 1% BSA (Fraction V Roche Diagnostic, Darmstadt, Germany) and 0.1% Tween 20 (Merck, Darmstadt, Germany), the slides were incubated overnight at 4 °C with the primary antibodies diluted 1:50 in the blocking buffer. Antibodies directed against the following proteins were used: Aurora B (mouse monoclonal ab-3609, Abcam, Cambridge, UK), pPLK1 (PLK1 pT210, rabbit monoclonal ab155095, Abcam), pericentrin (rabbit polyclonal ab4448, Abcam), centrin 1 (mouse monoclonal, clone 20H5, Merck Millipore, Burlington, MA, USA), and α-tubulin (mouse monoclonal, T6199, Sigma, Saint Louis, MO, USA). The slides were then washed with PBS and incubated for 1 h with the secondary antibody (diluted 1:500 in the blocking buffer, Alexa Fluor 488-conjugated goat anti-rabbit and Alexa Fluor 594-conjugated goat anti-mouse, Invitrogen, Carlsbad, CA, USA), washed with PBS, and mounted with mounting medium containing DAPI (4,6-diamidino-2-phenylindole, Invitrogen). Digital images of the cells were captured using a Nikon fluorescence microscope (Nikon Eclipse 90i, Melville, NY, USA) equipped with a CCD camera (DS-Qi1 Monochromatic Digital Camera, Melville, NY, USA). The digital images were analyzed for the immunostained proteins at specific locations and scored using Adobe Photoshop© (Adobe Photoshop CS6, Adobe system Inc. San Jose, CA, USA) and ImageJ (http://rsb.info.nih.gov, 12 April 2018) software. The distance between centrosomes placed in the same focused plane during mitosis was measured using NIS imaging software (NIS Elements, Nikon). The fluorescent signal intensity of pPLK1 (green fluorescence) in the digital images was quantified in the whole nuclear area (blue fluorescence) of a hundred cells using NIS software.

### 2.5. Metaphase Plate Analysis

The growing MEF Tet-FAK cells in ON and OFF conditions for three days were incubated with 30 ng/mL colcemid (KaryoMAX, Invitrogen, Waltham, MA, USA) for 90 min to induce metaphase arrest, washed in hypotonic buffer containing 75 mM KCl, fixed with methanol: acetic acid (3:1), dropped onto cold glass slides and mounted in the medium containing DAPI. The digital images were captured using the fluorescence microscope and analyzed using Adobe Photoshop software. Chromosome plates containing approximately 40 chromosomes were scored as diploid, whereas chromosome numbers close to the double and triple number, were scored as tetraploid and polyploid plates, respectively. At least 500 chromosome plates were analyzed for each condition.

### 2.6. SiRNA Transfection

Specific siRNA directed against human FAK (ON-TARGETplus SMARTpool siRNAs) and nontarget control were supplied by Dharmacon (Lafayette, CO, USA) and transfected with TransIT-X2 reagent (Mirus Bio LLC, Madison, WI, USA) into BJ fibroblast cells. The knock-down efficiency was checked by immune-blotting using a specific antibody against FAK as described below. 

### 2.7. Western Blotting

Total cell lysates were prepared in lithium dodecyl sulfate sample buffer (LDS, Novex, Life technologies, Carlsbad, CA, USA), fractionated in precast 4–12% SDS-PAGE gradient gels (Biorad, Mini-Protean-TGX, Hercules, CA, USA), and transferred to the nitrocellulose membrane (Thermo Scientific, Rockford, IL, USA). The blots were probed with primary antibodies, pPLK1 (1:1000, pT210, ab155095, Abcam, Cambridge, UK), pEg5 (1:1000, pSer1033 [38]), PLK1 (1:1000, 37-7000, Invitrogen, Carlsbad, CA, USA), pFAK (1:1000, pTyr397, 44624G, Invitrogen, Carlsbad, CA, USA), FAK (1:1000, 610087, BD Biosciences, San Diego, CA, USA), Cyclin B1 (1:1000, sc-245, Santa Cruz Biotechnology, Santa Cruz, CA, USA), and β-actin (1:5000, ab 6276-100, Abcam), followed by the appropriate HRP-conjugated secondary antibody (HRP-conjugated donkey anti-rabbit, NA9340V and HRP-conjugated sheep anti-mouse, NA9310V, GE Healthcare, Chicago, IL, USA) and developed by the enhanced chemiluminescence method (Amersham ECL, GE Healthcare, Chicago, IL, USA). The results were analyzed using Image Lab software (v4, Bio-Rad Laboratories, Hercules, CA, USA).

### 2.8. Statistical Analysis

In all the experiments, at least 50–100 randomly selected cells per condition and time point were analyzed in each of at least three independently repeated experiments (N = 3). The statistical analysis was performed using the student’s *t*-test. *p*-values less than 0.05 were considered as significant. *p* values less than 0.05, 0.01, 0.001, and 0.0001 were shown by one, two, three, and four stars, respectively. 

## 3. Results

### 3.1. Absence of Integrin-Mediated Cell Adhesion Prolongs G2 Phase and Delays Mitotic Progression in the Early Stages

Cell adhesion was previously found to affect PLK1 function during cytokinesis [18]. Therefore, in order to investigate whether the cell adhesion to ECM via integrins affects the PLK1-driven cell cycle progression during the G2 and M phases, the human non-transformed BJ fibroblast cells were first synchronized at the early S phase by the double thymidine block method. The cells were then trypsinized five hours after release from thymidine and reseeded on fibronectin- or PLL-coated dishes as the integrin-dependent and -independent adhesion conditions, respectively. The cell cycle progression through G2 and M phases was monitored by live-cell imaging after treating the cells with a specific DNA probe (SPY DNA) to easily visualize the cells in both culture conditions. When the adherent cells reached mitosis, they rounded up and the condensed chromosomal DNA emitted a sharp fluorescence signal appearing as an intensity peak, which disappeared once the DNA was decondensed (Figure S1A,B and Movie S1A,B). Thus, the start and end times of the individual intensity peaks determine the initiation of DNA condensation and decondensation during the late G2 and the telophase, respectively. When the DNA condensation was monitored in the PLL-attached cells, the intensity surface plot analysis showed a significantly increased length of both G2 phase and early mitosis in the cells re-plated on PLL, compared to the fibronectin plate (Figure S1C). 

**Figure 1:**
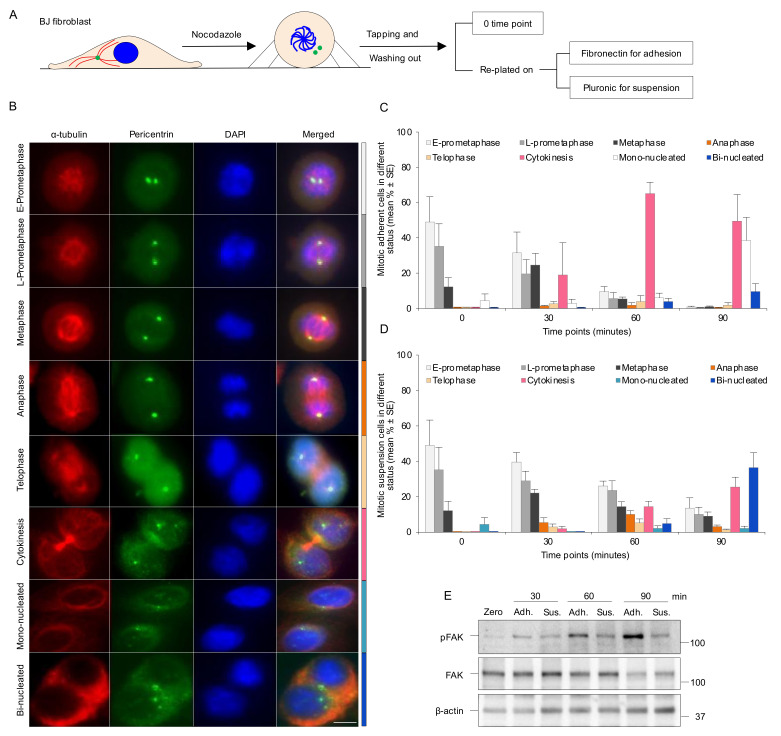
Absence of integrin-mediated cell adhesion causes a delay in the early mitotic progression. (**A**) Experimental design illustrating how the isolated mitotic cells were used to check mitotic progression in the different culture conditions. Centrosomes, microtubules, and nucleus/chromosomes are shown in green, red, and blue, respectively. (**B**) Representative immunofluorescence micrographs illustrating the mitotic progression of BJ cells from early (E)- and late (L)-prometaphase to cytokinesis, the presence of divided cells (mono-nucleated cells), and the cells that failed to complete cytokinesis (bi-nucleated cells). The centrosomes and the mitotic spindle were labeled with antibodies against pericentrin (green) and α-tubulin (red) directly after nocodazole washout (0 min time-point) and after a 30, 60, and 90 min incubation period of the cells adhering to fibronectin or kept in suspension. Nuclei were stained with DAPI. Scale bar, 10 μm. (**C**,**D**) Mean (%) ± SE of the number of adherent (**C**) and non-adherent cells (**D**) in different mitotic stages determined based on centrosomes’ location, spindle formation, and nucleus status as shown in part B. The color of each bar on the graph corresponds to the different mitotic phases shown in the side color bar of part B. (**E**) Western blot of the mitotic cells treated as in (**C**,**D**) to monitor the phosphorylation of FAK at Tyr397 and total FAK at the described time points.

To further analyze how the absence of adhesion causes early mitotic delay, the cells were synchronized in the prometaphase using nocodazole and the mitotic progression was chased after the release. The mitotic round cells were isolated by tapping the flask (shake-off), released into mitosis by washing for approximately 20 min, and reseeded into Pluronic (non-adherent)- or fibronectin (adherent)-coated dishes (Figure 1A). The progression through the mitotic stages was determined by the analysis of centrosomes’ location, bipolar spindle assembly, and chromosome distribution at different time points (Figure 1B). Staining of the cells with DAPI and antibodies against α-tubulin and the PCM protein pericentrin showed that at the 0 min time-point of reseeding, essentially all the cells were in the early stages of mitosis, demonstrating that the cell isolation procedure resulted in a highly synchronized cell population enriched at the beginning of the M phase (Figure 1C,D). After 60 min, the majority of mitotic adherent cells (>70%) reached or passed the cytokinesis stage (Figure 1C), whereas non-adherent cells were distributed in different stages at the same time point with 50% still remaining at prometaphase (Figure 1D). In agreement with our previous report [18], the adherent cells completed cytokinesis within 180 min, while non-adherent cells were unable to divide and accumulated as bi-nucleated cells (about 60%) or prometaphase cells at the later time points (Figure S2A,B). The fate of the latter cells was not followed, but prolonged prometaphase is likely to result in checkpoint slippage into G1 as tetraploid cells or cell death [39]. As expected [40], the level of activated FAK (pTyr397-FAK) was low at the 0 time-point (rounded mitotic cells) and remained low in the non-adherent cells as compared to the gradually increasing levels in the adhering cells after the drug washout (Figure 1E and Figure S2C).

To follow the nocodazole-synchronized cells in mitosis by live imaging, the isolated cells were reseeded on fibronectin- and PLL-coated plates. While almost all the cells on fibronectin flattened during anaphase and reached cytokinesis within one hour after synchronization release (Movie S2A), most of the mitotic cells on PLL remained rounded at the same time period (Movie S2B) and exhibited abnormal morphological features at the later time points (Figure S2D). These results confirm our previously reported observation [18] that adhesion to ECM is required to complete the late stage of cytokinesis, and in addition suggest that it has a role in promoting the progression through the early mitotic stages. 

### 3.2. Absence of Integrin-Mediated Cell Adhesion Causes Centrosome Abnormality

**Figure 2:**
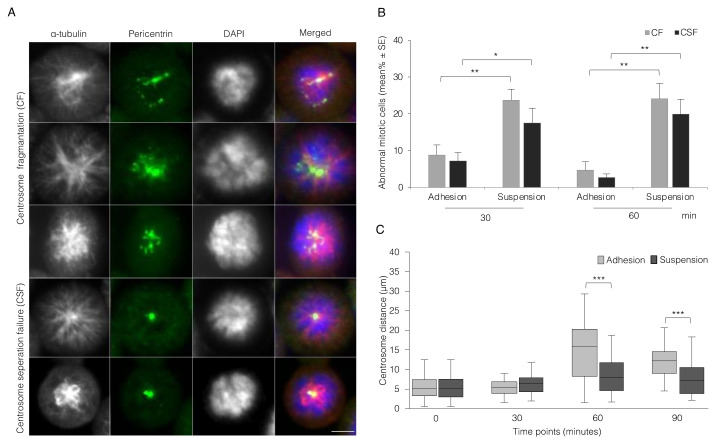
Lack of integrin-mediated cell adhesion causes centrosome abnormality. (**A**) Representative immunofluorescence images of mitotic cells containing centrosome-related abnormalities, found frequently in non-adherent cells: centrosome fragmentation (CF) and centrosome separation failure (CSF). The centrosomes and the mitotic spindle of BJ cells were labeled with antibodies 30 and 60 min after nocodazole release as described in the Figure 1A,B. Scale bar, 10 μm. (**B**) Mean (%) ± SE of the number of cells with CF and CSF present at the indicated time points. (**C**) Box plots showing the centrosomes separation rate by measuring the distance (µm) between the pericentrin-stained spindle poles (Figure S3) at the indicated time points after nocodazole washout. Only cells with two centrosomes and in the same microscope focused plane were analyzed here. *p*-values less than 0.05, 0.01, 0.001, and 0.0001 were shown by one, two and three stars, respectively.

During the analysis of mitotic progression described above, two types of centrosome abnormalities were observed in the non-adherent cells that could explain the observed early mitotic delay, i.e., centrosome fragmentation (CF) and centrosome separation failure (CSF) (Figure 2A). The number of cells having CF and CSF during the suspension culture reached close to 25 and 20%, respectively, after 60 min compared to 5 and 2% for the adherent cells (Figure 2B). Notably, the non-adherent cells often exhibited an abnormal mitotic spindle due to the close location of the centrosomes, even when they had a normal number of non-fragmented centrosomes. Measurement of the distance between the centrosomes located in the same focused plane (Figure S3) at different time points after nocodazole washout revealed a striking failure to normally separate centrosomes in the non-adherent cells as compared to the adherent cells (Figure 2C). 

### 3.3. FAK Activity Promotes G2 to M Transition and Progression in Early Mitosis

**Figure 3:**
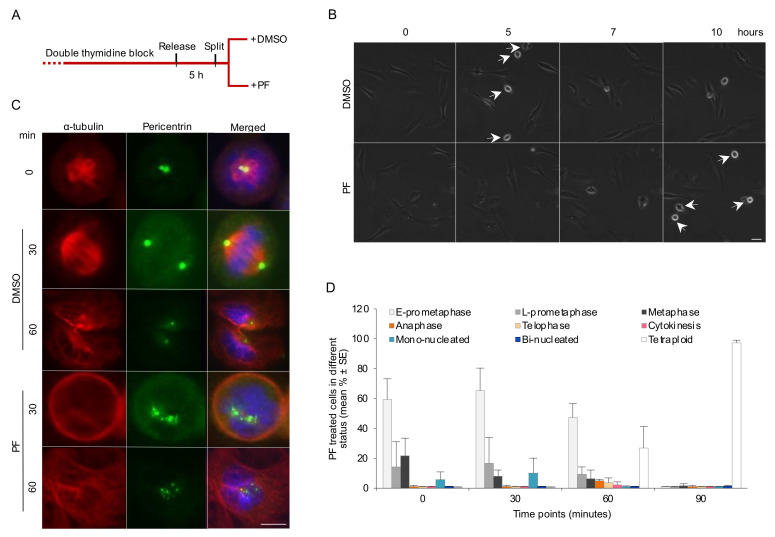
Inhibition of FAK activity slows down the G2 to M transition and impairs early mitotic progression. (**A**) Design of the experiment shown in B. (**B**) Snapshots from a representative time-lapse movie showing the cells at different time points after release from the thymidine block and in the presence of PF or DMSO as control. The arrows mark the cells rounding up for mitosis. Scale bar, 50 μm. (**C**) Representative immunofluorescence pictures showing the mitotic progression of BJ cells after nocodazole washout in the presence of PF or DMSO as a control. The centrosomes and the mitotic spindle were labeled with antibodies as described in Figure 1B. Scale bar, 10 μm. (**D**) Mean (%) ± SE of the number of PF-treated cells in different mitotic stages as described in Figure 1C.

To identify signaling events downstream of integrin-mediated cell adhesion supporting an error-free G2/M transition and early mitotic progression, we first examined the involvement of FAK, a non-receptor tyrosine kinase with a central role in integrin-associated signaling. Addition of the selective FAK inhibitor PF-562271 (PF) to fibronectin-adhered BJ cells after the double thymidine block release (Figure 3A) prolonged the duration of G2 compared to DMSO-treated cells as the control (Figure 3B and Movie S3A,B), similar to the previous result with the cells reseeded on PLL (Figure S1). The efficient inhibition of FAK activation by PF was confirmed by Western blotting of the cells after re-plating on fibronectin-coated dishes for 90 min (Figure S4A).

A detailed analysis of the mitotic progression in the presence of PF was performed using the same approach as described in Figure 1B. While most of the DMSO-treated adherent control cells were found in cytokinesis at the 60 min time-point after the nocodazole synchronization release, a large fraction of the PF-treated cells remained in prometaphase and had failed in the formation of a bipolar spindle at this time point, and approximately 90% of the cells never completed karyokinesis, and therefore became tetraploid mononuclear cells (Figure 3C,D). A large increase in the number of cells containing delocalized PCM was seen in the PF-treated cells (Figure 3C and Figure S4B). The possibility that this centrosome fragmentation was associated with centriole splitting or overduplication was analyzed by immunostaining of centrin 1 (Figure S4C). While PF caused abnormal distribution of pericentrin, the centrioles appeared as doublets comparable to centrioles under the control condition. 

### 3.4. FAK Inhibition Reduces PLK1 Activity and Thereby Impairs Centrosome Separation and Bipolar Spindle Assembly

**Figure 4:**
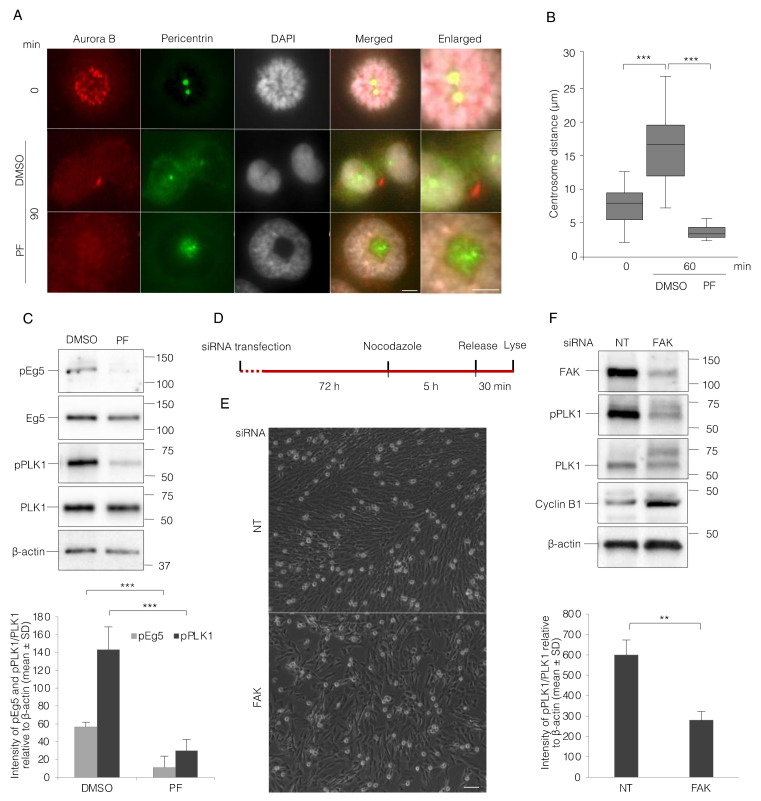
FAK inhibition reduces PLK1 activity and thereby impairs centrosome separation. (**A**) Representative immunofluorescence pictures illustrating the separation of centrosomes and nuclear features in BJ cells at the time points of 0 min after nocodazole washout and 90 min after re-plating the cells on fibronectin with PF or DMSO added directly at the washout. The staining of Aurora B (red) shows its location at centromeres and the midbody at the early and late stages of mitosis, respectively, in the control cells. Scale bar, 5 μm. (**B**) Box plots showing the centrosomes distance (µm) at 0 and 60 min after treatment as in (**A**). (**C**) Representative Western blot picture (upper) and quantification of the signal intensity of the bands (lower) of mitotic cells re-plated on fibronectin for 60 min in the presence of PF or DMSO. (**D**) Experimental design for siRNA treatment shown in (**E**,**F**). (**E**) Representative phase contrast images from the siRNA transfected cells 5 h after nocodazole treatment. Scale bar, 50 μm. (**F**) Western blot pictures (upper) and quantification of the signal intensity of pPLK1 bands relative to total PLK1 and β-actin (lower) from the cells described in part (**D**). *p*-values less than 0.05, 0.01, 0.001, and 0.0001 were shown by two and three stars, respectively.

We asked next if defective bipolar spindle formation is caused by centrosome separation failure in the absence of FAK activity. The cells were immunostained for pericentrin and Aurora B to clearly detect centrosomes and mitotic stages. After 90 min, most of the control cells had reached cytokinesis, but the PF-treated cells had formed a monopolar spindle phenotype (Figure 4A). The distance between the centrosomes was measured 60 min after the mitosis synchronization release (Figure 4B) and showed approximately a two-fold increase in the DMSO-treated cells, whereas it was even reduced in the PF-treated cells compared to the cells at the 0 min time-point (Figure 4B). Since PLK1 plays key roles during the entire G2 to M transition and mitotic progression, its activity together with its downstream indirect target Eg5, required for the centrosome separation, were checked by Western blotting after PF or DMSO treatment of the adherent cells. FAK inhibition for 60 min after the synchronization release clearly reduced the phosphorylation of PLK1 and Eg5 at Thr210 and Ser1033, respectively (Figure 4C). To directly test the requirement of PKL1 activity for the spindle formation during this time period of the cell cycle, the same experiment was repeated where the cells were treated with PLK1 inhibitor BI-6727. The PLK1 inhibitor induced a similar effect on centrosome separation and PCM (pericentrin) delocalization as PF. Furthermore, inhibition of Aurora A, which phosphorylates PLK1 at Thr210 in the activation loop, also prevented centrosome separation (Figure S5A,B).

To further analyze the relation of PLK1 status and FAK, the fibroblast BJ cells were transfected with FAK-directed and non-targeting (NT) siRNAs. The FAK protein level was significantly suppressed after 72 h in the FAK siRNA-treated cells, and nocodazole was then added for 5 h to enrich the number of cells in mitosis before the cells were lysed for the analysis by WB (Figure 4D). The nocodazole treatment resulted in an accumulation of mitotic rounded cells, indicating that the interphase cells were still proceeding in the cell cycle in both FAK and control siRNA-transfected cells (Figure 4E). Phosphorylation of PLK1 was significantly reduced in the FAK knock-down cells compared to NT controls (Figure 4F). This data confirms the above-described results with the FAK inhibitor, suggesting that the reduced PLK1 activity links FAK to the G2/M transition delay and also to the early mitotic progression failure. 

### 3.5. Lack of FAK Expression Resembles the Effect of Integrin-Mediated Adhesion on Mitotic Progression in MEF Cells 

**Figure 5:**
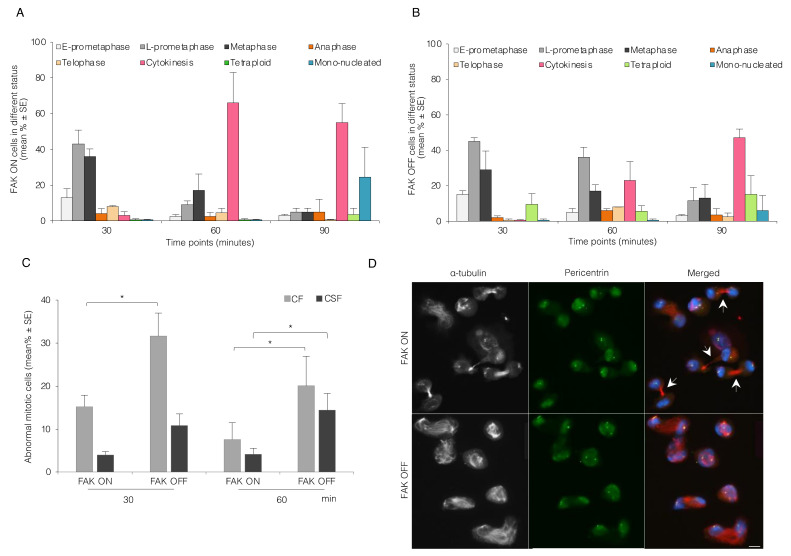
Inhibition of FAK expression in Tet-FAK MEF cells impairs centrosome separation and bipolar spindle formation. (**A**,**B**) Mean (%) ± SE of the number of Tet-FAK ON (A) and Tet-FAK OFF cells (**B**) in different mitotic stages as described in Figure 1B. (**C**) Mean (%) ± SE of the number of Tet-FAK ON and OFF cells containing abnormal centrosomes (CF and CSF) present at different mitotic stages. (**D**) Representative immunofluorescence pictures illustrating FAK ON and FAK OFF cells 60 min after release from mitotic block. The arrows point to the microtubule bundle of cytokinetic cells (FAK ON). Pericentrin (green), α-tubulin (red/white), and DAPI (blue). Scale bar, 10 μm. *p*-values less than 0.05, 0.01, 0.001, and 0.0001 were shown by one star, respectively.

As an alternative approach to test if FAK can regulate the events described above, we used Tet-FAK cells [36], a MEF cell line in which the expression of FAK is under the control of tetracycline. FAK expression is switched on in the absence of doxycycline and efficiently switched off by the addition of doxycycline (a tetracycline analog) for three days. These cells were nocodazole-synchronized at the M phase and analyzed as described above. The FAK OFF cells gave similar results as the BJ cells kept in suspension or treated with FAK inhibitor in all aspects, i.e., mitotic progression (Figure 5A,B), centrosome separation (Figure S6), and the induction of centrosome abnormalities (Figure 5C). Similar to BJ cells, the majority of mitotic MEF FAK ON cells had reached cytokinesis at the 60 min time-point after nocodazole release, whereas FAK OFF cells showed early mitotic delay and many cells contained abnormal centrosomes and abnormal microtubule spindle, which caused an irregular cell shape (Figure 5D). 

**Figure 6:**
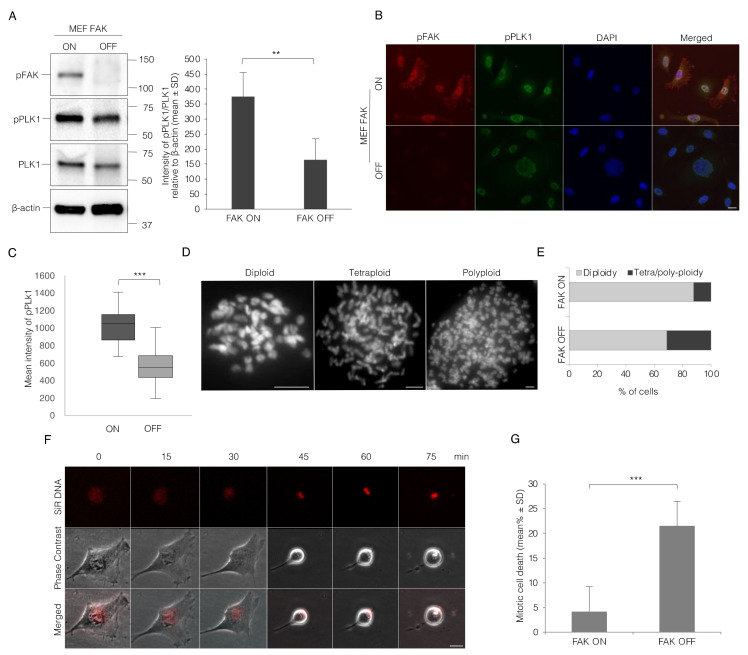
Inhibition of FAK expression reduces the activity of PLK1 and causes frequent mitotic cell death and tetraploidy. (**A**) Representative Western blot pictures and quantification of the bands showing the difference in the level of pPLK1 relative to the total level of PLK1 and β-actin, and pFAK in the mitotic Tet-FAK-ON and -OFF MEF cells re-plated on fibronectin for 60 min. (**B**). Decrease of pPLK1 in the cells lacking FAK expression after 72 h treatment with doxycycline and 5 h after release from the double thymidine block. Representative micrographs illustrating the pPLK1 (green) signals in Tet-FAK ON and OFF cells. Scale bar, 20 μm. (**C**) Box plots showing the average intensity of pPLK1 in 100 cell nuclei. (**D**) Representative pictures of Tet-FAK OFF cells with different ploidy. The chromosomes were stained by DAPI. Scale bar, 5 μm. (**E**) The % of mitotic cells having diploid or tetra/polyploid chromosome sets. A total of 500 chromosome plates were analyzed for each condition of FAK ON and OFF. (**F**) Snapshot images from a time-lapse movie displaying the mitotic progression of a single FAK OFF cell from the beginning of mitosis (0 min time point) to cell death at the later time point. The nucleus was labeled with SiR-DNA (red). Scale bar, 50 μm. (**G**) Mean (%) ± SD of the number of mitotic cells that die during the early mitotic progression after rounding up. *p*-values less than 0.05, 0.01, 0.001, and 0.0001 were shown by two and three stars, respectively.

Moreover, switching FAK expression off resulted in significantly reduced phosphorylation of PLK1 in the mitotic cells after 72 h from adding doxycycline as shown by Western blotting of whole culture lysates and by single cell analysis of immune staining intensity in the nucleus (Figure 6A–C). The effects of turning off FAK in these cells resembled to a large extent the effects of the PF treatment as well. To analyze whether the mitotic error after the short-term switching off of FAK induces abnormal ploidy, the cell cycle was blocked in the metaphase using colcemid treatment, and the number of chromosomes was counted in at least 500 cells. As expected, the population of tetra- and poly-ploid cells was raised (about 30%) compared to FAK ON cells (Figure 6D,E). In spite of the centrosome separation defect, live-cell imaging showed that most FAK OFF MEF cells continued to proliferate; however, numerous cells died during mitosis in each round of the cell cycle (Figure 6F,G and Movie S4).

## 4. Discussion

Our previous work has shown that integrin-induced signals are required for the completion of the late stage of cytokinesis by the recruitment of the ESCRT-III complex to the MB and the subsequent abscission [18]. Failed cytokinesis generates cells with four centrosomes in case they would proceed to the next mitosis, a situation resulting in multipolar mitotic spindle and chromosomal segregation defects. The integrin-dependent recruitment of ESCRT-III to the MB was linked to FAK and PLK1 [18]. Since PLK1 is a master regulator of several events during the G2 and M phases of the cell cycle, we investigated in this study whether integrin-mediated adhesion affected these stages of the cell cycle in addition to G1 to S transition and cytokinesis. For this purpose, non-transformed human fibroblasts and FAK knockout MEF cells were synchronized either at the S or the early M phases and the cell cycle progression was followed under the adherent and non-adherent conditions, and after the abrogation of FAK function.

In the absence of integrin-mediated adhesion, BJ cells were moderately delayed in the passage through the G2 phase. The transition into mitosis occurs when a critical level of active cyclin B-CDK1 has accumulated, a tightly regulated process where PLK1 has central roles. Severe problems were then observed in the separation of centrosomes, the subsequent formation of a normal bipolar mitotic spindle, and the progression from prometaphase to metaphase. Similar effects, but even more enhanced, were seen after treating the adherent cells with the FAK inhibitor (PF-562271); the transition from G2 to mitosis was delayed, centrosome separation was essentially blocked, and most of the cells became tetraploid due to failed karyokinesis. Identical results were obtained with two other FAK inhibitors, PND1186 and FAK inhibitor 14 (data not shown). Interestingly, activating phosphorylation of the centrosome-separating kinesin Eg5, as well as of the upstream kinase PLK1 [28], were reduced in the adherent cells treated by the FAK inhibitor. As expected from these data and in agreement with previous studies [41], pharmacological inhibition of PLK1 activity blocked the centrosome separation. The strong effects of the FAK and PLK1 inhibitors on centrosomes may be due to the fast and efficient action on the kinase activity, while the residual pTyr397-FAK seen in the non-adherent cell (Figure 1E) may explain the less drastic outcome. The residual pTyr397-FAK probably reflects the turnover rate after detachment, or alternatively it may represent a FAK pool activated by integrin-independent stimuli. 

In addition to FAK, the related kinase PYK is also activated by integrin-mediated adhesion and inhibited by PF. Thus, the results obtained from the non-adherent cells or PF-treated cells could be due to the lack of either FAK activity, PYK activity, or both. To clarify the contribution of FAK to the above results, we used the Tet-FAK cells. In these cells, the endogenous FAK gene is disrupted, and the FAK expression from the stably transfected construct can be efficiently turned off by the presence of doxycycline in the culture medium. The results from these cells under the conditions of FAK expression turned on or off show that FAK has a major role in promoting centrosome separation and progression to metaphase.

It was previously reported that a mutation in the cytoplasmic domain of β1 integrin subunit, which disturbs integrin activation and thereby ligand binding and signaling, caused the formation of a multipolar spindle. The chromosome segregation defect was suggested to generate bi-nucleated cells due to interference with cytokinesis [33]. Similarly, the deletion of FAK in primary endothelial cells was found to cause multipolar mitotic spindles and increased cell death [42]. However, in light of our later findings showing that integrin-mediated adhesion and FAK activity are required for specific steps in the late cytokinetic abscission process [18,43], it is clear that the interpretation of causes for and consequences of aberrant mitotic spindles after disrupted integrin signaling is complex. The presence of >2 centrosomes in the following cell cycle after a failed cytokinesis probably contributed to the spindle defects observed in the studies described above [18,32], thus complicating the analysis of the possible direct effects of integrin signals on centrosome separation and stability. The same problems are associated with the use of non-synchronized Tet-FAK OFF cells in our study. The spindle defects originating from either aberrant cytokinetic abscission or centrosomes separation and stability in the absence of FAK signals are affecting each other in a circular manner, resulting in some cells dying at mitosis in each round of the cell cycle and a surviving cell population, which is heterogeneous in chromosome and centrosome numbers. Since we were aware of this complication from our previous cytokinesis studies, the analysis of centrosome separation distance in Tet-FAK cells was done on cells having only two centrosomes with no or minor additional pericentrin-stained structures (the latter exemplified in Figure S6). Notably, according to our data, genomic heterogeneity is most likely an inherent property of all FAK-deficient cell lines, an understanding that should be considered in the evaluation of results obtained with such cells. Also, the Tet-FAK ON cell population contained cells with abnormal nuclei and centrosome numbers, but less frequently than Tet-FAK OFF cells. This heterogeneity is not surprising since it would have been generated already when the FAK-deficient MEFs were established [36], and after transfection with the FAK-coding plasmid, the most dysfunctional cells have presumably been competed out among the FAK expressing cells.

Our data shows that adhesion is necessary for centrosome separation through the activation of FAK and PLK1 and the following phosphorylation of Eg5 at Ser1033 [28]. Together with Thr926 phosphorylation by CDK1, the modification of Eg5[Ser1033] has been shown to result in the translocation of the kinesin motor to the vicinity of centrosomes, which is necessary for centrosome separation during prophase [28]. Our findings thus identify a mechanism by which cell adhesion can regulate spindle formation. However, the linking steps between integrin/FAK and PLK1 are unclear. The activation of PLK1 requires phosphorylation at Thr210 by Aurora A, and additional modifications by PAK1 and Src have been reported to also be of importance for PLK1 activity [44,45]. While the latter kinases are known mediators in FAK signaling pathways, such a connection has not been reported for Aurora A. The regulation of Aurora A is complex and several different activation mechanisms have been elucidated [46], including binding to Ser112-phosphorylated Bora, autophosphorylation at Thr288 after interactions with Tpx2, and transphosphorylation after interactions with Cep192 [47]; furthermore, PAK1 was shown to promote Aurora A Thr288 phosphorylation [48]. Thus, further investigations are needed to clarify how FAK regulates PLK1 activity, possibly via Aurora A.

During the transient period of cell rounding, pTyr397 and other pY sites in FAK are dephosphorylated and instead FAK becomes phosphorylated on several Ser residues, including Ser732 [42]. Interestingly, pS732-FAK has been shown to localize at the spindle microtubules and to promote the formation of a normal spindle by stimulating the dynamic microtubule turnover [49]. This kinase-independent function of FAK may thus follow after the FAK kinase-dependent stimulation of centrosome separation. 

In addition to the impact on centrosome migration and spindle formation, centrosome disruption was also seen in a significant fraction of the cells in the absence of adhesion and FAK activity during early mitosis. Such disruptions may be due to dissociation of the PCM material from the centrosome by the altered cytoskeletal forces, suggested to be promoted by low PLK1 and CDK1 activity [50,51], or by altered unknown protein modifications of PCM components. While PCM fragmentation can also result from proteolytic cleavage by separase [52], this was apparently not the case under the conditions of our study, since the presence of Sepin-1 (separase inhibitor) did not rescue the fragmentation in the non-adherent cells (data not shown). Fragmentation of the single interphase centrosome was previously described to occur in astrocytes lacking the β1 integrin subunit, as well as in WT astrocytes (β1+/+) treated with the myosin II inhibitor Blebbistatin or kept in suspension [35]. In the latter study, FAK was found to not be required for the stability of the interphase centrosome, suggesting that it is regulated in different ways to the mitotic centrosomes in our study.

## 5. Conclusions

The present study identified novel roles of integrin-mediated adhesion for the cell cycle regulation during mitosis, in addition to the previously known regulation of the G1-S transition and the cytokinetic abscission. Here, the adhesion is shown to also promote centrosome segregation and their structural integrity at the beginning of mitosis. FAK, PLK1, and Eg5 are implicated as signaling intermediates linking integrins to the formation of a bipolar mitotic spindle and error-free chromosome distribution.

## Data Availability

Not applicable.

## Supplementary Materials

The following are available online at https://www.mdpi.com/article/10.3390/cells11081360/s1, Figure S1: Absence of integrin-mediated cell adhesion prolongs G2- and early M-phase duration, Figure S2: The number of adherent and non-adherent cells in different mitotic stages at the extended time points, Figure S3: The separation of centrosomes is impaired in the non-adherent cells, Figure S4: FAK inhibition causes PCM delocalization, Figure S5: Centrosomes separation failure and PCM fragmentation in BJ cells after inhibition of PLK1, FAK, or Aurora A, Figure S6: The separation of centrosomes in the Tet-FAK-ON and -OFF cells, Movie S1A,B: The cell cycle progression of BJ fibroblasts during G2 and M phases, Movie S2A,B: The lack of adhesion causes mitotic progression delay, Movie S3A,B: FAK inhibition prolongs the G2 phase, Movie S4: Induction of mitotic cell death after switching off FAK.

## References

[1] Geiger B., Bershadsky A., Pankov R., Yamada K.M. (2001). Transmembrane crosstalk between the extracellular matrix—Cytoskeleton crosstalk. Nat. Rev. Mol. Cell Biol..

[2] Acebron I., Righetto R.D., Schoenherr C., de Buhr S., Redondo P., Culley J., Rodríguez C.F., Daday C., Biyani N., Llorca O. (2020). Structural basis of Focal Adhesion Kinase activation on lipid membranes. EMBO J..

[3] Mitra S.K., Hanson D.A., Schlaepfer D.D. (2005). Focal adhesion kinase, in command and control of cell motility. Nat. Rev. Mol. Cell Biol..

[4] Gough R.E., Jones M.C., Zacharchenko T., Le S., Yu M., Jacquemet G., Muench S.P., Yan J., Humphries J.D., Jørgensen C. (2021). Talin mechanosensitivity is modulated by a direct interaction with cyclin-dependent kinase-1. J. Biol. Chem..

[5] Jones M.C., Askari J.A., Humphries J.D., Humphries M.J. (2018). Cell adhesion is regulated by CDK1 during the cell cycle. J. Cell Biol..

[6] Jones M.C., Zha J., Humphries M.J. (2019). Connections between the cell cycle, cell adhesion and the cytoskeleton. Philos. Trans. R. Soc. Lond. B. Biol. Sci..

[7] Lock J.G., Jones M.C., Askari J.A., Gong X., Oddone A., Olofsson H., Göransson S., Lakadamyali M., Humphries M.J., Strömblad S. (2018). Reticular adhesions are a distinct class of cell-matrix adhesions that mediate attachment during mitosis. Nat. Cell Biol..

[8] Marchesi S., Montani F., Deflorian G., D’Antuono R., Cuomo A., Bologna S., Mazzoccoli C., Bonaldi T., Di Fiore P.P., Nicassio F. (2014). DEPDC1B coordinates de-adhesion events and cell-cycle progression at mitosis. Dev. Cell.

[9] Dix C.L., Matthews H.K., Uroz M., McLaren S., Wolf L., Heatley N., Win Z., Almada P., Henriques R., Boutros M. (2018). The Role of Mitotic Cell-Substrate Adhesion Re-modeling in Animal Cell Division. Dev. Cell.

[10] Dao V.T., Dupuy A.G., Gavet O., Caron E., de Gunzburg J. (2009). Dynamic changes in Rap1 activity are required for cell retraction and spreading during mitosis. J. Cell Sci..

[11] Moreno-Layseca P., Streuli C.H. (2014). Signalling pathways linking integrins with cell cycle progression. Matrix Biol..

[12] Sambandamoorthy S., Mathew-Steiner S., Varney S., Zuidema J.M., Gilbert R.J., Van De Water L., LaFlamme S.E. (2015). Matrix compliance and the regulation of cytokinesis. Biol. Open.

[13] Zhu X., Ohtsubo M., Bohmer R.M., Roberts J.M., Assoian R.K. (1996). Adhesion-dependent cell cycle progression linked to the expression of cyclin D1, activation of cyclin E-cdk2, and phosphorylation of the retinoblastoma protein. J. Cell Biol..

[14] Shanmugasundaram K., Block K., Nayak B.K., Livi C.B., Venkatachalam M.A., Sudarshan S. (2013). PI3K regulation of the SKP-2/p27 axis through mTORC2. Oncogene.

[15] Brunet A., Bonni A., Zigmond M.J., Lin M.Z., Juo P., Hu L.S., Anderson M.J., Arden K.C., Blenis J., Greenberg M.E. (1999). Akt promotes cell survival by phosphorylating and inhibiting a Forkhead transcription factor. Cell.

[16] Hognas G., Tuomi S., Veltel S., Mattila E., Murumagi A., Edgren H., Kallioniemi O., Ivaska J. (2012). Cytokinesis failure due to derailed integrin traffic induces aneuploidy and oncogenic transformation in vitro and in vivo. Oncogene.

[17] Mierzwa B., Gerlich D.W. (2014). Cytokinetic abscission, molecular mechanisms and temporal control. Dev. Cell.

[18] Kamranvar S.A., Gupta D.K., Huang Y., Gupta R.K., Johansson S. (2016). Integrin signaling via FAK-Src controls cytokinetic abscission by decelerating PLK1 degradation and subsequent recruitment of CEP55 at the midbody. Oncotarget.

[19] Bastos R.N., Barr F.A. (2010). Plk1 negatively regulates Cep55 recruitment to the midbody to ensure orderly abscission. J. Cell Biol..

[20] Petronczki M., Lenart P., Peters J.M. (2008). Polo on the Rise-from Mitotic Entry to Cytokinesis with Plk1. Dev. Cell.

[21] Roshak A.K., Capper E.A., Imburgia C., Fornwald J., Scott G., Marshall L.A. (2000). The human polo-like kinase, PLK, regulates cdc2/cyclin B through phosphorylation and activation of the cdc25C phosphatase. Cell Signal..

[22] Van Vugt M.A., Bras A., Medema R.H. (2004). Polo-like kinase-1 controls recovery from a G2 DNA damage-induced arrest in mammalian cells. Mol. Cell.

[23] Conduit P.T., Wainman A., Raff J.W. (2015). Centrosome function and assembly in animal cells. Nat. Rev. Mol. Cell Biol..

[24] Lee K., Rhee K. (2011). PLK1 phosphorylation of pericentrin initiates centrosome maturation at the onset of mitosis. J. Cell Biol..

[25] Nigg E.A., Holland A.J. (2018). Once and only once, mechanisms of centriole duplication and their deregulation in disease. Nat. Rev. Mol. Cell Biol..

[26] Mardin B.R., Agircan F.G., Lange C., Schiebel E. (2011). Plk1 controls the Nek2A-PP1gamma antagonism in centrosome disjunction. Curr. Biol..

[27] Tanenbaum M.E., Medema R.H. (2010). Mechanisms of centrosome separation and bipolar spindle assembly. Dev. Cell.

[28] Bertran M.T., Sdelci S., Regue L., Avruch J., Caelles C., Roig J. (2011). Nek9 is a Plk1-activated kinase that controls early centrosome separation through Nek6/7 and Eg5. EMBO J..

[29] Vitre B.D., Cleveland D.W. (2012). Centrosomes, chromosome instability (CIN) and aneuploidy. Curr. Opin. Cell Biol..

[30] Lens S.M.A., Medema R.H. (2019). Cytokinesis defects and cancer. Nat. Rev. Cancer.

[31] Toyoshima F., Nishida E. (2007). Integrin-mediated adhesion orients the spindle parallel to the substratum in an EB1- and myosin X-dependent manner. EMBO J..

[32] Petridou N.I., Skourides P.A. (2014). FAK transduces extracellular forces that orient the mitotic spindle and control tissue morphogenesis. Nat. Commun..

[33] Reverte C.G., Benware A., Jones C.W., LaFlamme S.E. (2006). Perturbing integrin function inhibits microtubule growth from centrosomes, spindle assembly, and cytokinesis. J. Cell Biol..

[34] Colello D., Mathew S., Ward R., Pumiglia K., LaFlamme S.E. (2012). Integrins regulate microtubule nucleating activity of centrosome through mitogen-activated protein kinase/extracellular signal-regulated kinase kinase/extracellular signal-regulated kinase (MEK/ERK) signaling. J. Biol. Chem..

[35] Peng H., Ong Y.M., Shah W.A., Holland P.C., Carbonetto S. (2013). Integrins regulate centrosome integrity and astrocyte polarization following a wound. Dev. Neurobiol..

[36] Owen J.D., Ruest P.J., Fry D.W., Hanks S.K. (1999). Induced focal adhesion kinase (FAK) expression in FAK-null cells enhances cell spreading and migration requiring both auto- and activation loop phosphorylation sites and inhibits adhesion-dependent tyrosine phosphorylation of Pyk2. Mol. Cell Biol..

[37] Fox M.H., Read R.A., Bedford J.S. (1987). Comparison of synchronized Chinese hamster ovary cells obtained by mitotic shake-off, hydroxyurea, aphidicolin, or methotrexate. Cytometry.

[38] Rapley J., Nicolas M., Groen A., Regue L., Bertran M.T., Caelles C., Avruch J., Roig J. (2008). The NIMA-family kinase Nek6 phosphorylates the kinesin Eg5 at a novel site necessary for mitotic spindle formation. J. Cell Sci..

[39] Vakifahmetoglu H., Olsson M., Zhivotovsky B. (2008). Death through a tragedy, mitotic catastrophe. Cell Death Differ..

[40] Yamakita Y., Totsukawa G., Yamashiro S., Fry D., Zhang X., Hanks S.K., Matsumura F. (1999). Dissociation of FAK/p130(CAS)/c-Src complex during mitosis, role of mitosis-specific serine phosphorylation of FAK. J. Cell Biol..

[41] Smith E., Hegarat N., Vesely C., Roseboom I., Larch C., Streicher H., Straatman K., Flynn H., Skehel M., Hirota T. (2011). Differential control of Eg5-dependent centrosome separation by Plk1 and Cdk1. EMBO J..

[42] Park A.Y., Shen T.L., Chien S., Guan J.L. (2009). Role of focal adhesion kinase Ser-732 phosphorylation in centrosome function during mitosis. J. Biol. Chem..

[43] Gupta D.K., Du J., Kamranvar S.A., Johansson S. (2018). Tension-induced cytokinetic abscission in human fibroblasts. Oncotarget.

[44] Maroto B., Ye M.B., von Lohneysen K., Schnelzer A., Knaus U.G. (2008). P21-activated kinase is required for mitotic progression and regulates Plk1. Oncogene.

[45] Liu X., Zheng H., Li X., Wang S., Meyerson H.J., Yang W., Neel B.G., Qu C.K. (2016). Gain-of-function mutations of Ptpn11 (Shp2) cause aberrant mitosis and increase susceptibility to DNA damage-induced malignancies. Proc. Natl. Acad. Sci. USA.

[46] Du R., Huang C., Liu K., Li X., Dong Z. (2021). Targeting AURKA in Cancer, molecular mechanisms and opportunities for Cancer therapy. Mol. Cancer.

[47] Tavernier N., Sicheri F., Pintard L. (2021). Aurora A kinase activation, Different means to different ends. J. Cell Biol..

[48] Zhao Z.S., Lim J.P., Ng Y.W., Lim L., Manser E. (2005). The GIT-associated kinase PAK targets to the centrosome and regulates Aurora-A. Mol. Cell.

[49] Rea K., Sensi M., Anichini A., Canevari S., Tomassetti A. (2013). EGFR/MEK/ERK/CDK5-dependent integrin-independent FAK phosphorylated on serine 732 contributes to microtubule depolymerization and mitosis in tumor cells. Cell Death Dis..

[50] Wang G., Chen Q., Zhang X., Zhang B., Zhuo X., Liu J., Jiang Q., Zhang C. (2013). PCM1 recruits Plk1 to the pericentriolar matrix to promote primary cilia disassembly before mitotic entry. J. Cell Sci..

[51] Oshimori N., Ohsugi M., Yamamoto T. (2006). The Plk1 target Kizuna stabilizes mitotic centrosomes to ensure spindle bipolarity. Nat. Cell Biol..

[52] Karki M., Keyhaninejad N., Shuster C.B. (2017). Precocious centriole disengagement and centrosome fragmentation induced by mitotic delay. Nat. Commun..

