# Supplementary material for: Integrin-Mediated Adhesion Promotes Centrosome Separation in Early Mitosis
Source: Cells. 2022 Apr 16;11(8):1360. doi: 10.3390/cells11081360 (PMC9030014; doi:10.3390/cells11081360)
Supplement: Supplementary file 1 [file cells-11-01360-s001.zip › Revised figures and movies-2022-02-13-2/cells-1424884supp.pdf]

Supplementary figures

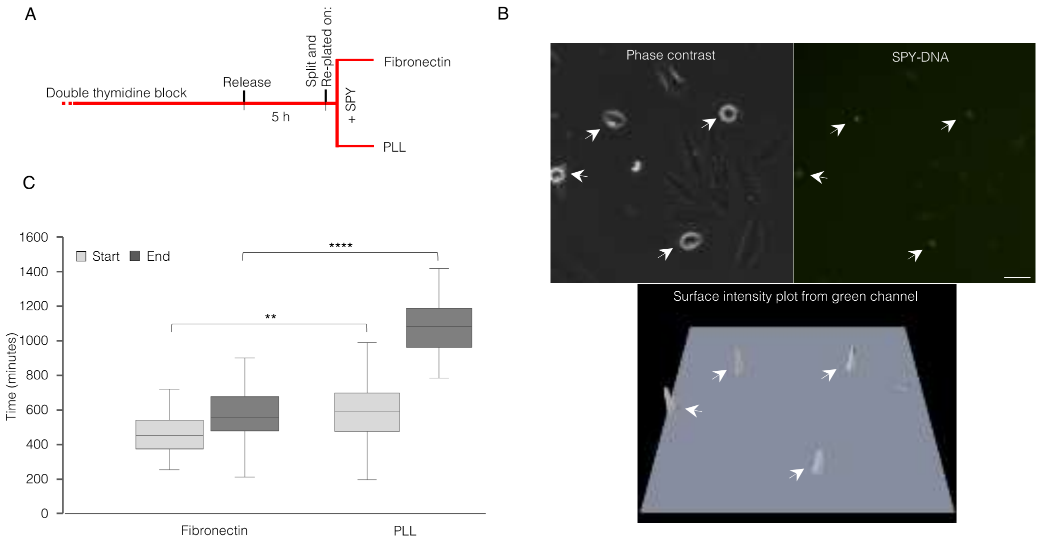

Figure S1. Absence of integrin-mediated cell adhesion prolongs G2- and early M-phase duration. (A) Experimental design illustrating 5 hours incubation after double thymidine release followed by trypsinization (split) and addition of the SPY probes before the start of 24 hours time-lapse recording. (B) A representative snapshot from the time-lapse movies showing the mitotic cells (arrows), recognized by rounded morphology (phase contrast) and condensed chromosomes (SPY-DNA, green), which matches the individual peaks on the surface intensity plot of the green channel. (C) Box plots showing start and end time points of the DNA intensity peaks in the cells re-plated on fibronectin- or PLL-coated plates. For each condition, at least 50 cells were counted. Scale bar, 50 μm. p-values less than 0.01, and 0.0001, were shown by two and four stars, respectively.

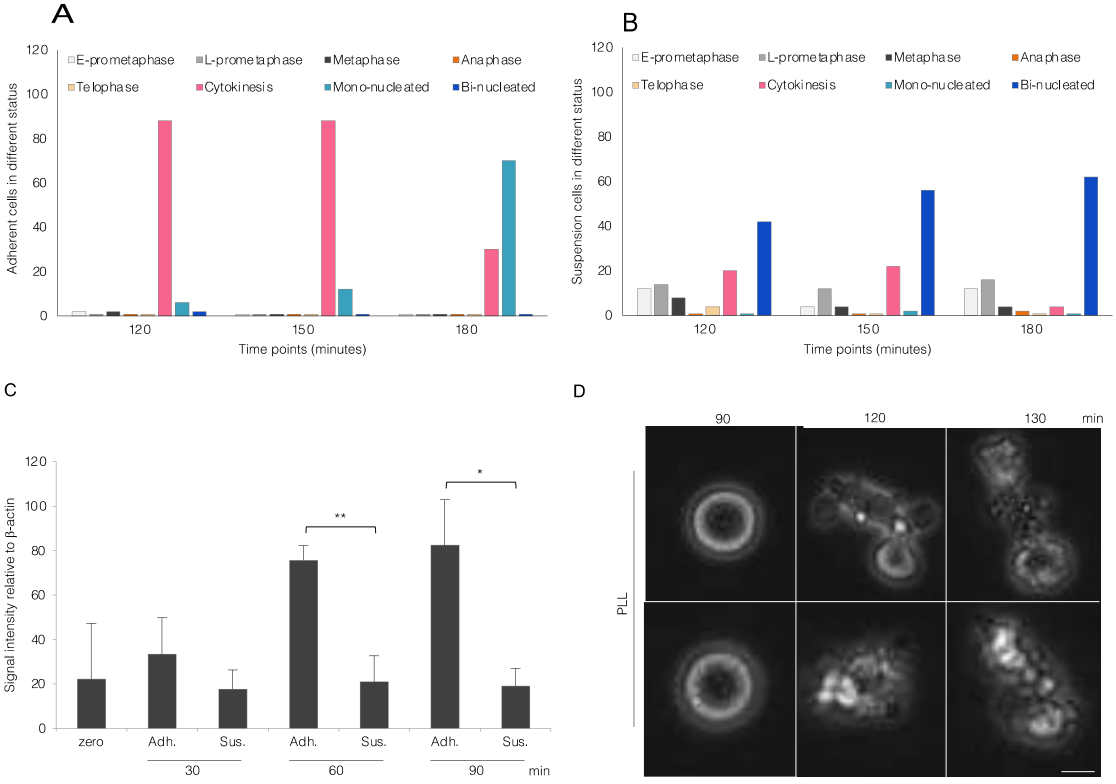

Figure S2. The number of adherent and non-adherent cells in different mitotic stages at the extended timepoints.

(A) and (B) Mean (%) of the number of adherent- (A) and non-adherent- (B) cells in different mitotic stages determined as described in Fig 1B, after incubation of the cells adhering to fibronectin or kept in suspension for the indicated extended time periods. The representative results of one out of 4 independent experiments are shown. (C) The quantification of signal intensity of pTyr397-FAK western blot bands relative to β-actin shown in Fig 1E. (D) Representative snapshots from Movie 2B at the different time points. Scale bar, 10 µm. p-values less than 0.05, 0.01, were shown by one, and two stars.

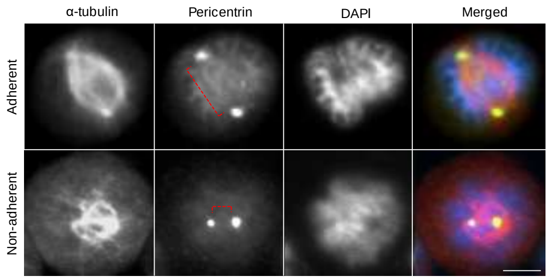

Figure S3. The separation of centrosomes is impaired in the non-adherent cells. Representative images showing how the centrosomes distance was measured between the pericentrin-stained spindle poles in the adherent and non-adherent cells. The cells with only two centrosomes were selected for this analysis. Scale bar, 10 μm.

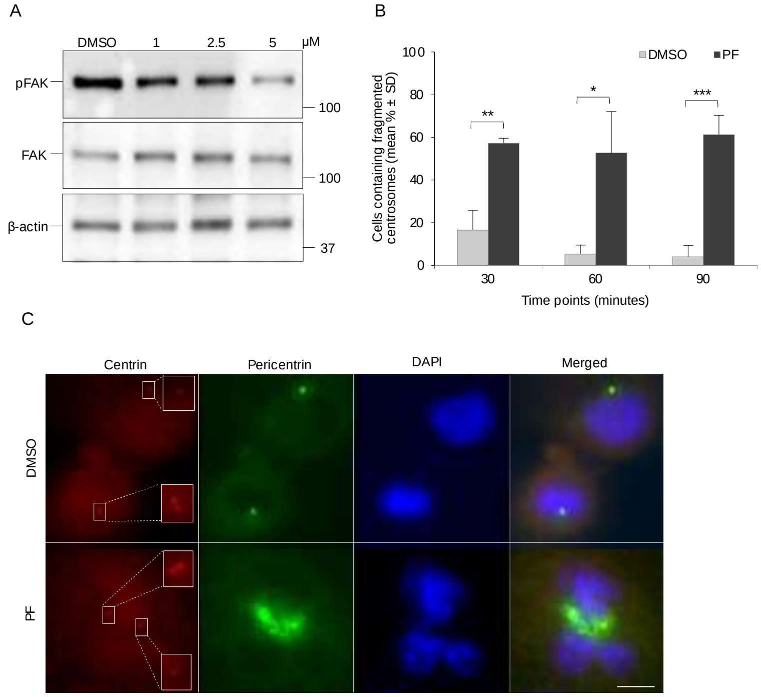

Figure S4. FAK inhibition causes PCM delocalization. (A) Western blot of nocodazole-synchronized mitotic cells re-plated on fibronectin for 90 minutes showing the level of FAK inhibition (pTyr397-FAK) in the presence of DMSO or PF at different concentrations. (B) Mean (%) ± SD of PF and DMSO-treated cells containing the localized PCM (pericentrin). (C) Representative immunofluorescence micrographs illustrating centrioles (centrin, red), PCM (pericentrin, green) and DNA (blue) of mitotic BJ cells treated with PF or DMSO as a control after synchronization release for 60 minutes. Scale bar, 10 μm. p-values less than 0.05, 0.01, and 0.001 were shown by one, two and three stars, respectively.

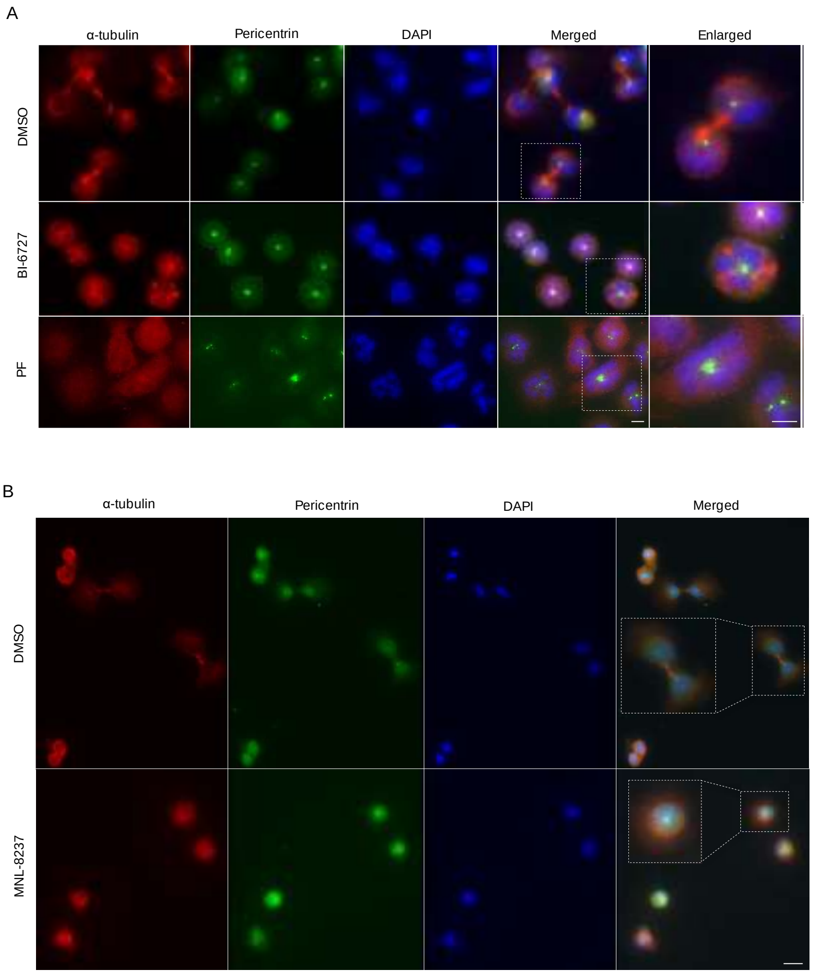

Figure S5. Centrosomes separation failure and PCM fragmentation in BJ cells after inhibition of PLK1, FAK, or Aurora

A. Representative immunofluorescence micrographs showing the mitotic progression of nocodazole-synchronized BJ cells after 60 minutes in the presence of BI-6727, PF-562271 (PF) (A), MNL-8237 (B) or DMSO. The centrosomes and the mitotic spindle were labeled with antibodies as described in the Fig 1B. The enlarged images are from the square areas on the merged images. Scale bar, 5 μm (A) and 20 μm (B).

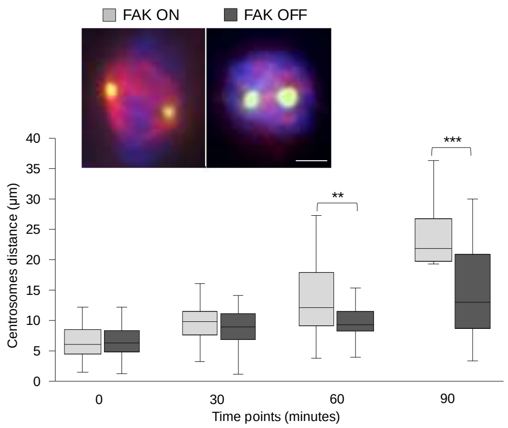

Figure S6. The separation of centrosomes in the Tet-FAK ON and OFF cells. The separation of centrosomes in the Tet-FAK-ON and -OFF cells were analyzed by measuring the distance between the center of pericentrin-stained spindle poles at the indicated time points after nocodazole washout and replating on fibronectin. The cells having more than 2 pericentrin-stained structures were excluded from this analysis, and one example each of immunostained Tet-FAK ON and OFF cells with two centrosomes are shown. Scale bar, 10 μm. p-values less than 0.01, and 0.001 were shown by two and three stars, respectively.

Movie S1A and B. The cell cycle progression of BJ fibroblasts during G2 and M phases. The movies show the cell cycle progression of BJ fibroblasts re-plated on (A) fibronectin- or (B) PLL-coated plates five hours after release from thymidine block. The images are split into channels of phase contrast, green (SPY DNA), together with a surface signal intensity plot of the green channel.

Movie S2A and B. The lack of adhesion causes mitotic progression delay. The movies show the mitotic progression of BJ cells re-plated on (A) fibronectin- or (B) PLL-coated plates after nocodazole release. The arrows show the cells that fail in normal mitotic progression.

Movie S3A and B. FAK inhibition prolongs the G2 phase. The time-lapse movies are chasing the synchronized BJ cells 5 hours after release from thymidine block in the presence of DMSO and PF inhibitor.

Movie S4. Induction of mitotic cell death after switching off FAK. The movie monitors the fate of FAK OFF MEF cells in culture after switching off FAK for three days. The arrows show the cells dying during mitosis after the rounding up. The dead cells are stained brightly red by Sir-DNA due to their disrupted plasma membranes, and most of them float off into the medium after death.
